# Chemically tailored carbon nanotubes as a new toolbox for biomedicine and beyond

**DOI:** 10.1042/BIO04104010

**Published:** 2019-08

**Authors:** Benjamin Barnes, Alexandra Brozena, YuHuang Wang

**Affiliations:** University of Maryland, USA

## Abstract

Like molecules of DNA, carbon nanotubes (CNTs) can display a variety of structures, but all conduct electrons and feature unique optical properties. In this perspective article, we highlight several recent works that bridge these two seemingly distant worlds. We illustrate the largely untapped potential of CNTs for biological research by exploring several developing biomedical applications utilizing nanotube semiconductors, including field effect transistor biosensors that couple high sensitivity with selectivity, and fluorophores for deep-tissue imaging whose excitation and emission wavelengths can be tuned throughout the near-IR II window simply by using defect chemistry.

Semiconducting CNTs may seem exotic to scientists working in biological systems, but these nanomaterials are in fact not as far removed from cellular components as they may initially appear. CNTs are a nanoscopic allotrope of carbon, the elemental backbone of all living things. Composed of a hexagonal lattice of carbon atoms rolled into a cylinder of ~1 nm in diameter and ~0.5–1 μm in length, these materials have dimensions that are on the order of actin microfilaments, DNA and selfassembled viral particles (Figure 1).

Furthermore, as in biological systems, the structure of a CNT dictates its properties. Specifically, the optoelectronic properties of a particular nanotube species (i.e., its allowable energy levels and number of electrons those levels can host) depend on the orientation of the hexagonal lattice with respect to the nanotube axis, which defines the nanotube’s ‘chirality’ or crystal structure. As semiconductors, these materials also exhibit important phenomena that may provide crucial functionality for biological applications, including environment-dependent electrical conductivity stemming from the field effect and bright fluorescence in the secondary near-IR window (NIR-II) (wavelengths of 1000–1350 nm). These two phenomena combined in a nanoscale, all-carbon package provide biologists with a unique but underexploited tool with great potential for sensing and imaging in various biological systems and processes.

Nanotubes are typically grown in a chemical vapour deposition process that results in a mixture of different chiralities displaying distinct electrical and optical properties. However, thanks to their biologically comparable length scale and structure–property relationship, purification techniques that have been traditionally used in biology, such as density gradient ultracentrifugation, liquid chromatography and counter-current chromatography, can be used to isolate a single species of nanotube. Some chiralities have no bandgap, meaning the CNT behaves as a metal nanowire, but most chiralities have a non-zero bandgap of 0.5–2 eV. This subset of materials exhibits optical and electronic properties common to other semiconducting nanomaterials, namely photoexcitation and a variable electrical conductivity. The primary advantage of CNTs over traditional metal oxide semiconductors is their carbon lattice, which favours interaction with biological systems. A striking example of this is the ability of single-stranded DNA (ssDNA) molecules to spontaneously wrap around a nanotube in a helical fashion.

## A tube-in-a-tube for electrical biosensing

Almost since their discovery, researchers have considered CNTs for potential biological applications, including electrical-based sensing, in which most efforts have aimed at improving the signal transduction and chemical selectivity. Single-walled CNTs can interact directly with a wide range of biomolecules, including DNA and proteins. When charged macromolecules approach the surface of a nanotube, there may be measurable changes in its conductivity due to two factors: scattering of the charge carriers as a result of physical contact with the analyte and modulation of the charge carrier population by the field effect. Therefore, creating a circuit from a semiconducting single-walled CNT and submersing it in a biological matrix yields an excellent sensor in terms of signal transduction. However, such devices have poor selectivity, meaning they cannot differentiate between different molecules with similar structures.

To solve the problem of selectivity, it is possible to introduce analyte-specific probes onto the nanotube surface that favour interactions with a particular molecule or species. For example, diazonium chemistry can be used to integrate a wide range of aryl-linked functional groups (such as carboxylic acids, amines and halides) on a CNT surface. Carbodiimide chemistry can then be used to attach an oligonucleotide or polypeptide probe through the available carboxylic acid. Devices made from such surface-modified tubes have successfully served as single-molecule probes in studying DNA hybridization, changes in protein conformation and enzyme kinetics. However, covalent modification of the CNT surface with such molecular probes disrupts the nanotube’s conductive aromatic network, which reduces the magnitude of the electrical signal. Because of this, there has traditionally been a trade-off between the density of probes on the nanotube surface and the electrical signal that the device can output. If the electrical signal is too low, the device is not suitable for clinical applications.

**Figure 2.**
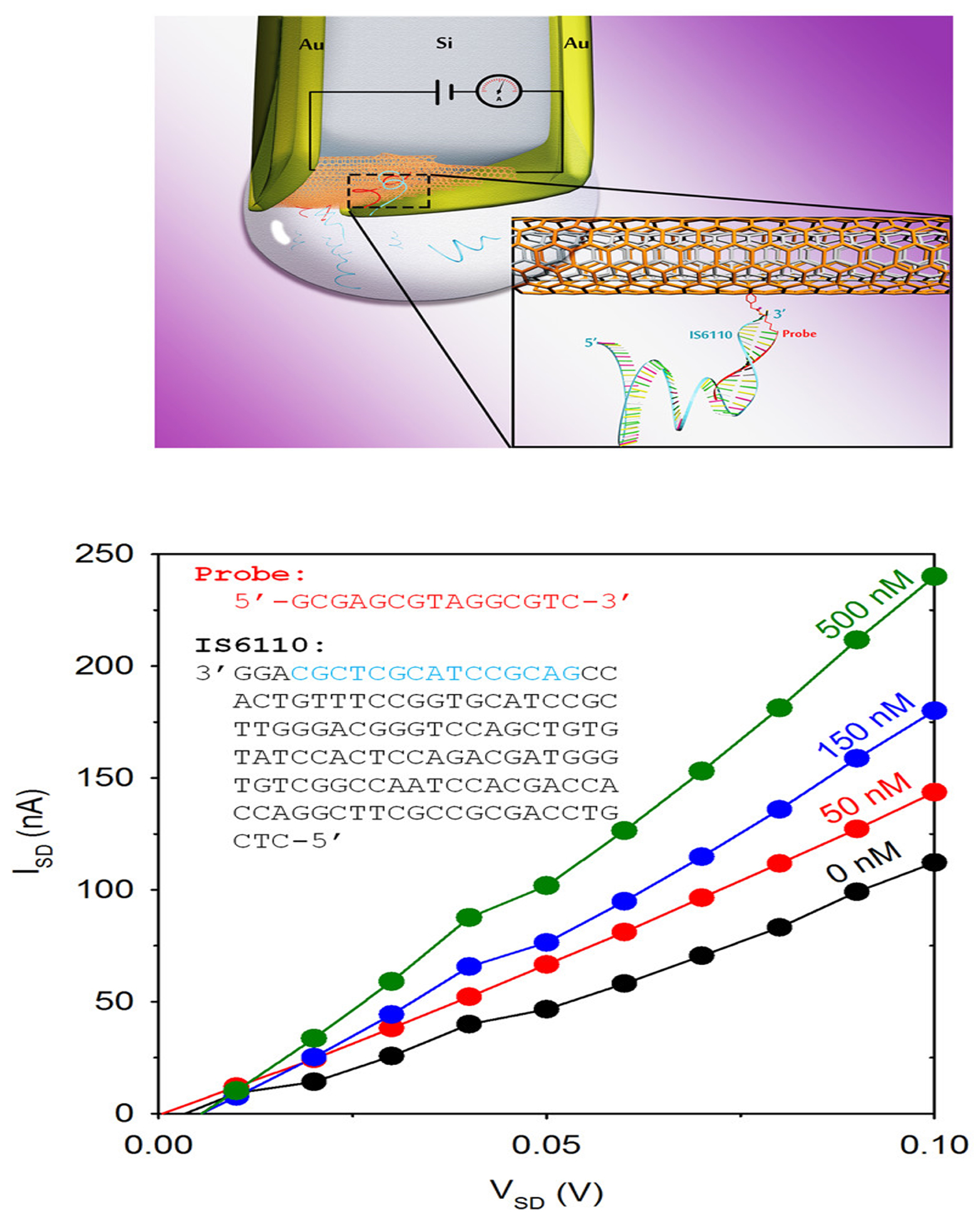
A Tubê2 structure enables electrical detection of biomolecules based on field effects using a simple device design with two electrodes. The third ‘gate’ electrode seen in other sensors is not required for Tubê2 sensors making them easier to fabricate. The nested structure of the Tubê2 allows the outer tube to be functionalized with receptors for high selectivity, while the inner tube’s electronic properties are preserved for signal transduction (https://pubs.acs.org/doi/10.1021/jacs.6b12111; Chemical gating of a synthetic tubein-a-tube semiconductor. (2017) JACS. **139**, 3045–3051).

In our lab, the conflicting needs for both signal transduction and selectivity in electrical sensing have largely been reconciled by using a tube-in-a-tube structure (what we call a Tubê2 semiconductor). Tubê2 semiconductors are synthesized from double-walled CNTs, which consist of two nanotubes nested within each other. The Tubê2 semiconductor is synthesized by loading the wall of the outer tube with a high density of covalently attached receptors. This provides the selectivity required in biosensing without compromising the electrical properties of the inner tube, which becomes electrically decoupled from the outer tube. This guarantees excellent signal transduction for sensing applications (Figure 2).

The electrical conductivity of the inner tube is modulated by the distribution of ions near the surface of the outer tube due to the field effect. We have shown that this Tubê2 semiconductor is excellent even for detecting small, charged analytes, such as ammonium ions, as well as biological macromolecules, such as ssDNA. To demonstrate the ability to detect relevant disease biomarkers, Tubê2 sensors were used to detect an oligonucleotide from the genome of the *Mycobacterium tuberculosis* bacterium, the vector for tuberculosis. A short, complementary probe for the bacterial DNA was attached to the surface of the Tubê2 using carbodiimide chemistry and benzoic acid functional groups implanted via diazonium chemistry (Figure 2). When the bacterial DNA docked with the probes on the Tubê2 sensor, there was a large increase in the conductivity of the Tubê2, indicating a positive diagnosis. This Tubê2 probe was also able to detect concentrations of the biomarker as low as 5 nM. Additionally, the high selectivity of the Tubê2 ssDNA probe enabled the differentiation of the target analyte from a nearly identical sequence featuring a single-base mismatch. Such differentiation is needed in clinical settings to prevent false-positive diagnoses that may arise from the presence of DNA that has a base sequence similar to the biomarker.

## Nanotubes as tunable fluorescent probes

**Figure 3.**
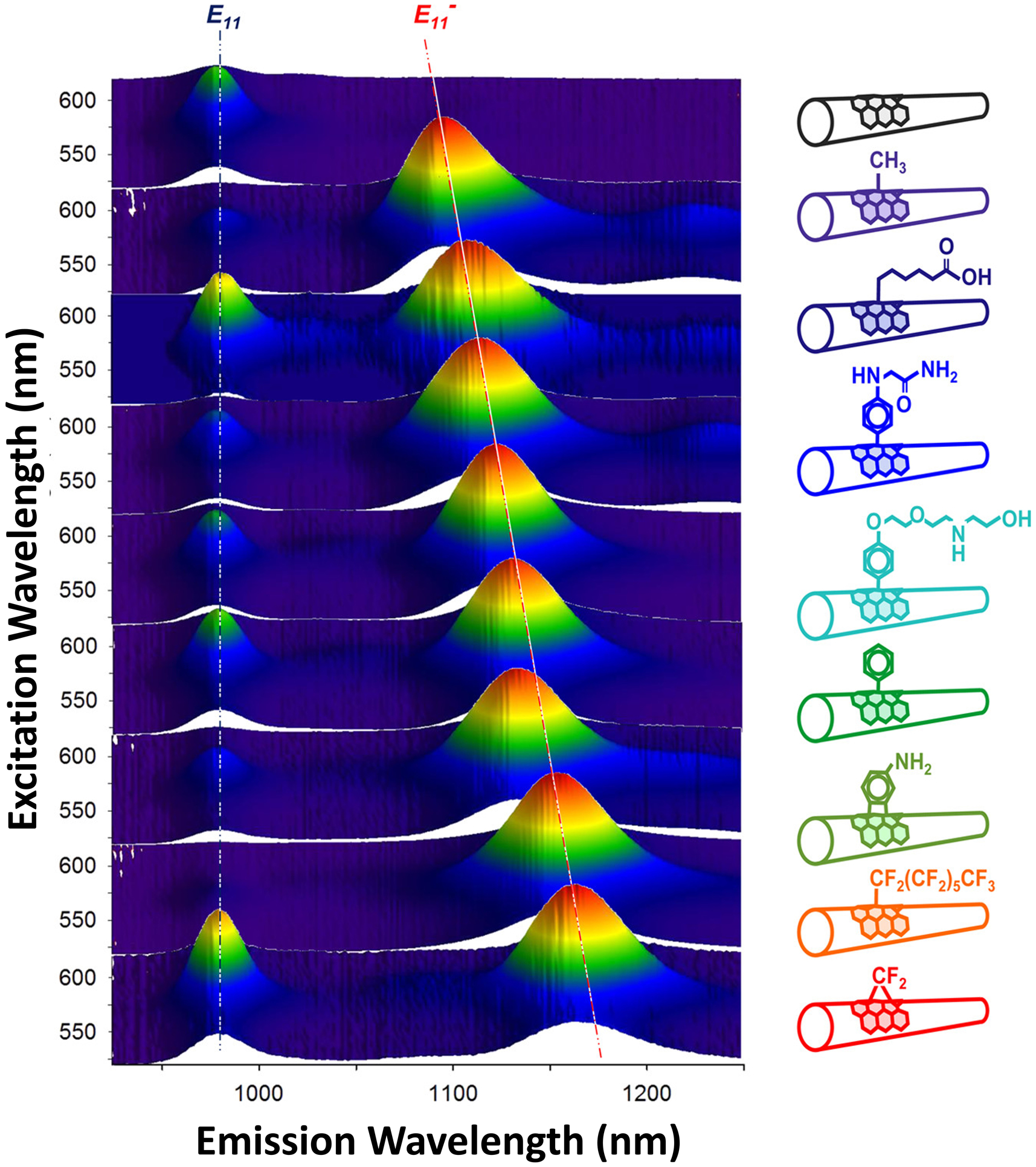
Fluorescent quantum defects hosted on single-walled CNTs show bright photoluminescence that can be tuned across the NIR-II window suitable for bioimaging (https://pubs.acs.org/doi/10.1021/jacs.6b03618; Molecularly tunable fluorescent quantum defects. (2016) JACS. **138**, 6878–6885).

Microbiology and biochemistry have developed in tandem with microscopy techniques, including those based on fluorescence, which have enabled the study of cells and tissues in unprecedented detail. These imaging techniques require fluorophores with well-defined excitation and emission wavelengths, but these are not always available, particularly in the NIR-II window, a region of the electromagnetic spectrum with maximum penetration depth for biological tissue. Fluorophores active in this region can potentially enable high-resolution imaging of deep tissues and organs. While many different fluorophores exist for a diverse range of samples, a tunable fluorophore that can be used across a wide range of wavelengths is especially desirable. Semiconducting CNTs also offer promise in this area as they absorb UV and visible photons to generate a sharp emission peak in the NIR, denoted as the E_11_ peak (Figure 3).

As with the bandgap and UV-visible spectrum of CNTs, the position of the E_11_ peak is determined by the nanotube’s structure. As a result, a given E_11_ peak position can be selected by purifying an individual nanotube species from a mixture of CNTs. The flexibility of this peak position is limited, however, by the requirement for techniques to purify nanotubes of a particular type/structure, the yield of the purification method and the relative population of that particular nanotube type in the raw material. Therefore, it is necessary to more precisely tune the emission peaks of a particular nanotube to a given range of wavelengths for imaging applications.

**Figure 4.**
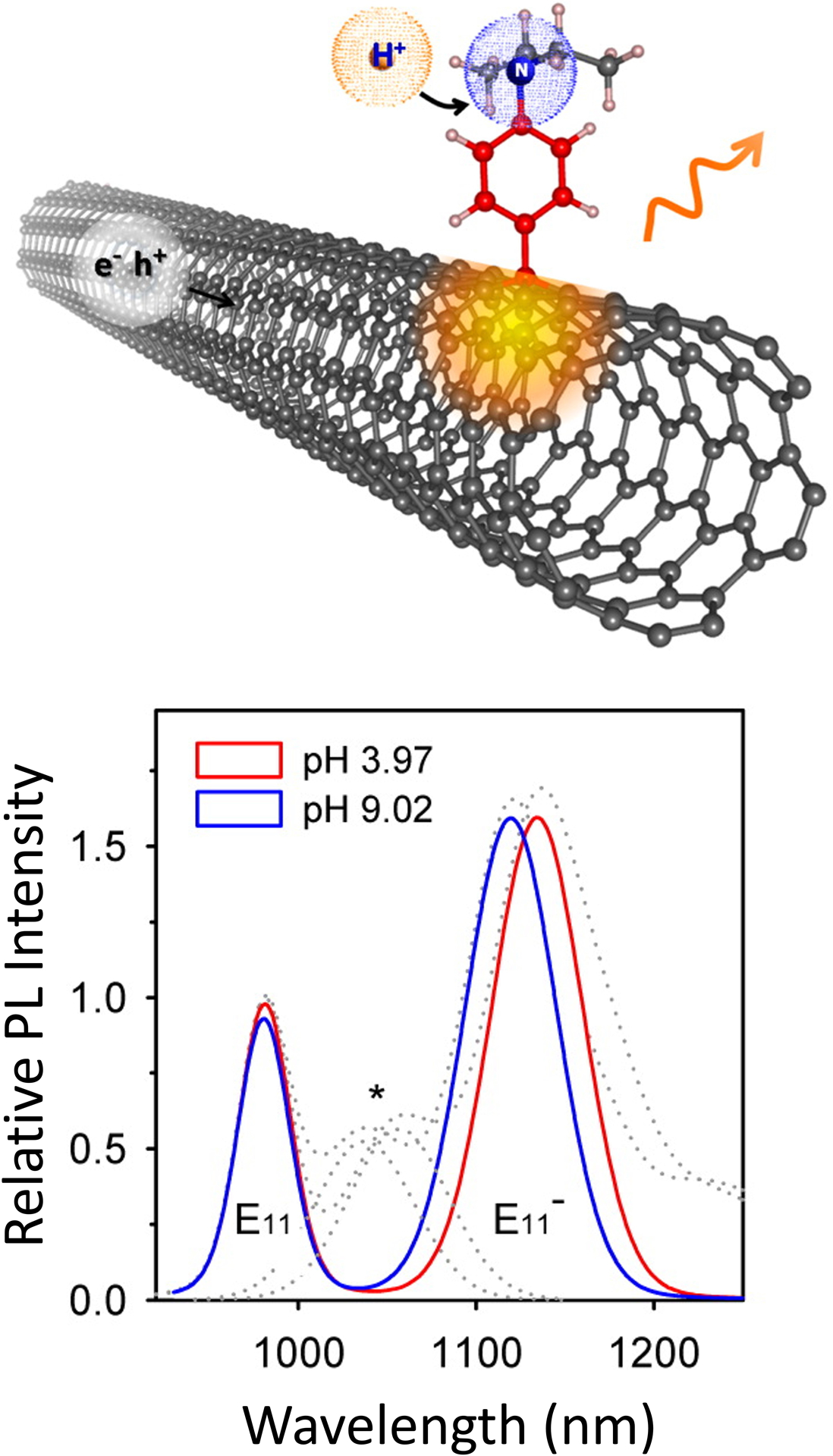
An optical pH meter: protonation of an N-containing defect centre leads to a large spectral shift in the shortwave infrared (J. Phys. Chem. C (2015) **119**, 7, 3733–3739; https://doi.org/10.1021/jp509546d).

Recently, our lab discovered that covalently attached functional groups (i.e., purposefully added ‘quantum defects’ in the nanotube structure) can create local variations in the nanotube’s energy that can trap mobile excitons and increase their probability of light emission, yielding a new, bright, defect photoluminescence (E_11_^−^) peak at a wavelength greater than the E_11_ emission of the pristine nanotube host (Figure 3). By varying the nanotube host and the types of molecules integrated on its surface, it has become possible to obtain fluorophores that can be tuned continuously across much of the NIR spectrum (Figure 3). Additionally, the defect emission can be modulated by the chemical environment if a defect is chosen that can selectively respond to chemicals, such as protons (Figure 4). This intersection of sensing and imaging can potentially be used to explore the chemical composition of biological systems at unprecedented resolution.

One promising application of this tunable defect emission is *in vivo* tissue imaging—a role traditionally considered unviable for CNTs due to toxicity and bioretention concerns and low photoluminescence brightness. However, it has recently been shown that ultrashort CNTs (< 100 nm) can be excreted from the body, thereby reducing the risk of bioretention. This has traditionally posed a problem, as fluorescence is not typically observed in ultrashort CNTs, because the mobile excitons become quenched at the nanotube ends. By introducing fluorescent quantum defects, we have shown that it is possible to create fluorescent ultrashort nanotubes that fluoresce brightly in the shortwave IR. This is a promising step in the practical exploitation of CNT fluorophores in tissue imaging.

## Conclusion

CNT semiconductors can be chemically tailored through the incorporation of fluorescent quantum defects into the sp^2^ carbon lattice and high-density loading of surface recognition groups in a Tubê2 semiconductor, to enable simultaneous sensitivity and ultrahigh selectivity in optical imaging and electrical detection. These chemically tailored carbon semiconductors have provided a versatile toolbox to probe the biological world.

## Figures and Tables

**Figure 1.**
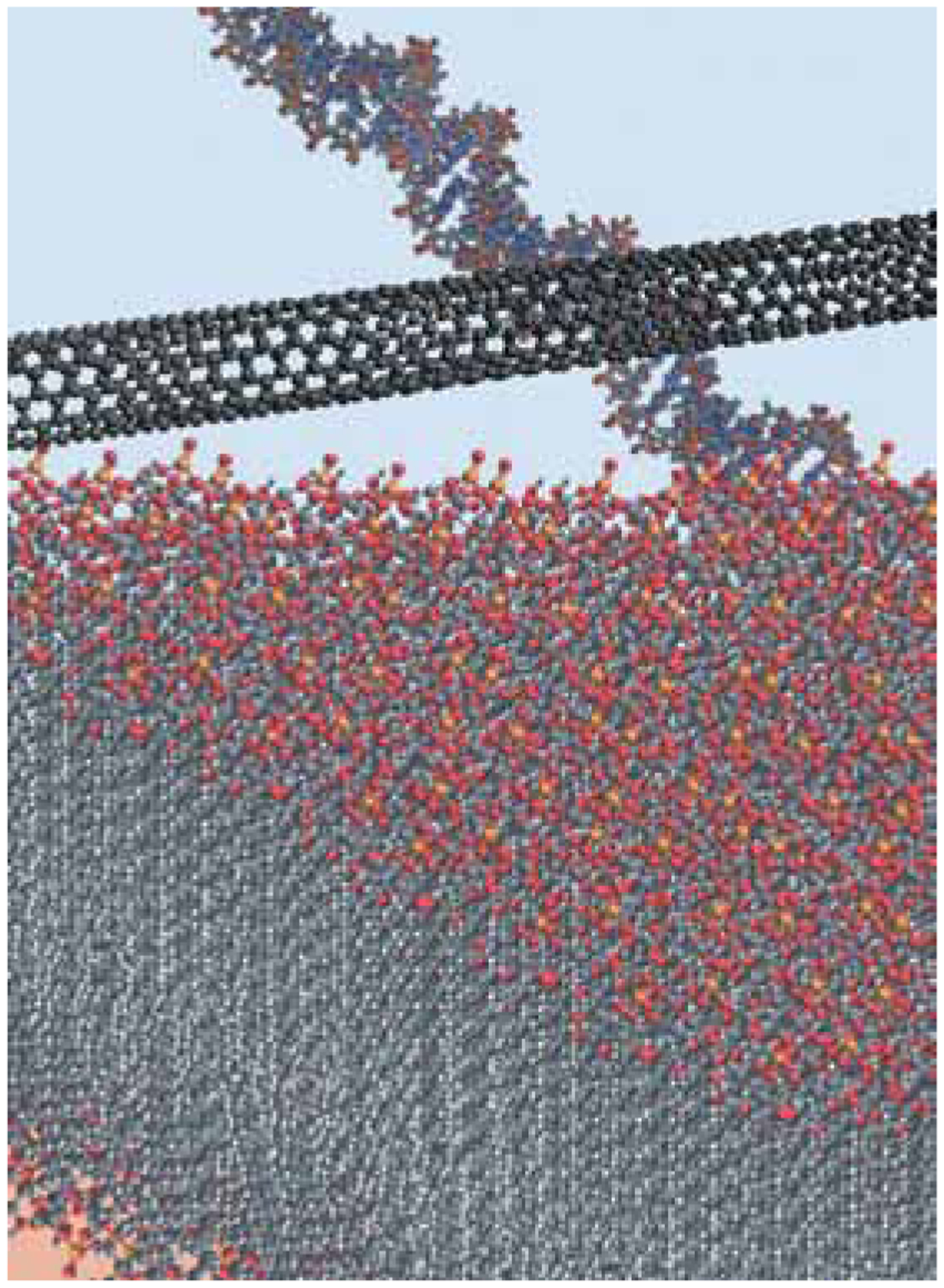
Nanotubes are of a comparable length scale to many familiar biological elements. A semiconducting single-walled CNT is shown here with a plasma membrane cross-section and a DNA segment. The similar length scale facilitates the use of CNTs as probes for such biological systems.
